# Analysis of topological relationships and network properties in the interactions of human beings

**DOI:** 10.1371/journal.pone.0183686

**Published:** 2017-08-23

**Authors:** Ye Yuan, Xuebo Chen, Qiubai Sun, Tianyun Huang

**Affiliations:** 1 School of Electronics and Information Engineering, University of Science and Technology Liaoning, Anshan, Liaoning, People’s Republic of China; 2 Graduate School, University of Science and Technology Liaoning, Anshan, Liaoning, People’s Republic of China; 3 School of Business Administration, University of Science and Technology Liaoning, Anshan, Liaoning, People’s Republic of China; 4 Center for Engineering Science and Advanced Technology, Peking University, Beijing, People’s Republic of China; Lanzhou University of Technology, CHINA

## Abstract

In the animal world, various kinds of collective motions have been found and proven to be efficient ways of carrying out some activities such as searching for food and avoiding predators. Many scholars research the interactions of collective behaviors of human beings according to the rules of collective behaviors of animals. Based on the Lennard-Jones potential function and a self-organization process, our paper proposes a topological communication model to simulate the collective behaviors of human beings. In the results of simulations, we find various types of collective behavior and fission behavior and discover the threshold for the emergence of collective behavior, which is the range five to seven for the number of topology *K*. According to the analysis of network properties of the model, the in-degree of individuals is always equal to the number of topology. In the stable state, the out-degrees of individuals distribute around the value of the number of topology *K*, except that the out-degree of a single individual is approximately double the out-degrees of the other individuals. In addition, under different initial conditions, some features of different kinds of networks emerge from the model. We also find the leader and herd mentality effects in the characteristics of the behaviors of human beings in our model. Thus, this work could be used to discover how to promote the emergence of beneficial group behaviors and prevent the emergence of harmful behaviors.

## Introduction

It is well known that collective motion phenomena exist widely in our world. For instance, flocks of birds and schools of fishes can move in relatively coordinated ways, especially when predators are approaching. The relatively coordinated movements (e.g., spiral locomotion) allow these kinds of collectively moving animals to resist predators effectively (e.g., groups of puffins utilize spiral locomotion to protect their nestlings from seagulls). In addition, various kinds of collective motion can also be found in systems of biomolecules, microbes and human beings. Thus, it is fascinating work for scientists from different fields to discover the principles of interactions among individuals in these systems and the relationship between local interactions and global emergences.

Collective motion can also be classed as a broad concept called collective behavior [1,2]. The collective behaviors of groups of animals [3,4], neurons [5–8] and human beings [9–12] have been widely discussed. In [13], Vicsek defined features of collective behavior: the action of an individual unit is dominated by the influence of its neighbors, and various ordering phenomena emerge from the system as the individuals simultaneously change their behavior to a common pattern. The main reason why a large number of individuals in the system can exhibit collective behavior is the interactions among them. Therefore, many scholars construct agent-based model to analyze the interactions among individuals in various kinds of systems. Specifically, two kinds of interactions among individuals have been widely studied: the interactive relationships of metric distance and topological distance. Under the rule of the interactive relationship of metric distance, every individual has a perception radius and can only communicate with other individuals within the range of the perception radius. Each individual is able to obtain information from other individuals in the process of interactions and will change its behavior according to the information received regarding the opinions and behavior of other individuals. Individuals in the group constantly change their behavior via this mechanism, and from the whole group, some characteristics of group behavior will emerge (e.g., collective behavior or fission behavior) at the macro level eventually. However, under the rule of the interactive relationship of topological distance, each individual does not need a perception radius and pays more attention to its nearest neighbors. Compared with the interactive relationship of metric distance, each individual has a larger perception range. The constraint on its perception ability is the number of nearest neighbors that it is able to communicate with. This number is called the number of topology in our paper. Each individual can obtain information from nearest neighbors regardless of how far away the neighbors are, and the number of these nearest neighbors is equal to the predefined number of topology. The main difference between these two methods is how the perception ability of individuals in the model is restricted. One is based on the range of the perception radius, and the other is based on the number of nearest neighbors, which are the neighbors that they can perceive.

Since the rules of interactions can be used to explain various phenomena in animals, bacteria and human beings, these two kinds of interactions have played a central role in the studies of collective behavior. For instance, many papers [3,4,14–21] demonstrate that the rules of metric distance can describe the interactions among individuals in various kinds of systems, and a variety of types of collective motion patterns can emerge based on the rules of metric distance. However, another kind of interactive relationship called topological distance has been analyzed in depth by scholars from various areas [22–25]. More precisely, in [22], Ballerini et al. found that each bird interacts with a fixed number of neighbors, rather than with all neighbors within a fixed metric distance, and the rules of topological interaction are more suitable to explaining the phenomena of collective behavior of birds.

The phenomenon that many individuals in the system can emerge the state of coherent motion and form characteristic shapes is caused by a self-organization process mentioned in [26]. The self-organization process illustrates how the pattern of a system at the global level emerges from the interactions among the units in the system at the local level; that is, the interactions among individuals give rise to the behavior of the whole group, which is quite different from the various behaviors of individuals. Self-organization processes are widely used to explain phenomena in the worlds of animals and bacteria, but less so for human beings. However, we note that a self-organization process exists in the collective activity of human beings.

In this paper, we construct a topological communication model based on a self-organization process and try to explain the interactions among human beings. To be specific, the interactions among different individuals in our model are a kind of topological interaction. The evolutionary process in our model is precisely a self-organization process. We add some changes to the traditional interactions among individuals mentioned above, and we also add some new elements to our model, which can make the spiral collective motion emerge. In this way, the model is enriched by the increased complexity. In addition, as topological relationships have been widely researched using the theory of complex networks [27–29], we also analyze and discuss the model from this perspective to discover the rules hidden in it, which may help us explain the rules of interactions in the behavior of human beings rather than other creatures.

## Methods

The spiral is a ubiquitous feature of the natural world and is found in living systems great and small–from the double helix structure of DNA to the shape and growth of animals and plants (e.g., conches and aloes). In addition, satellite images of hurricanes, the Fibonacci spiral in galaxies, and spiral waves in the neuronal system and fish migration are all natural phenomena that exhibit a spiral shape. We discover that spiral collective motion is an interesting phenomenon able to help us find some objective laws in the world. Thus, we attempt to construct a model of collective motion that can be used to implement our ideas.

First, we are inspired by some papers. For instance, a famous agent-based model of collective motion was introduced by Reynolds [3]. In his model, three rules were proposed: cohesion, separation and alignment. Based on these rules, collective phenomena successfully emerged from local interactions of agents. In addition, a simple rule that only includes a velocity-based alignment was put forward by Vicsek [30], and collective phenomena still emerged even though the rule was changed.

Second, different from [30], in our model, velocity-based alignment is ignored, and we seek to discover how the collective behavior emerges at the macroscopic level under a rule that only considers repulsion and attraction. Repulsion and attraction can change the positions of individuals and simultaneously change the distance between two individuals. This distance is different from the Euclidean distance used in traditional agent-based models. Under the background of interactions of human beings, we need to redefine the notions of metric distance and collision avoidance.

In the model, individuals tend to keep an expected distance from neighbors when the system is in a stable state, and collision avoidance exists in the process of interaction among individuals. These phenomena remind us of the “porcupine’s dilemma” proposed by German philosopher Arthur Schopenhauer. The story tells of the struggle of a group of porcupines that huddle to find warmth on a winter’s day. Similar to the porcupines, human beings also learn to keep an expected distance from one another, just enough to still enjoy the benefits of social interaction. To achieve the goal that every individual keeps an expected distance from every other, individuals continuously adjust the distance from neighbors under the effects of attraction and repulsion. It is impossible that one person provides all of his information in the process of interacting with other people because people are not willing to share unnecessary information (e.g., privacy and secrets) with others. Instead, each person prefers to give the amount of information to another person that is sufficient to guarantee that both of them can reach a consensus as expected. Similarly, people adjust the distance with others by changing the amount of information given to others, and this distance may be similar to the concept “psychological distance” mentioned in [31]. That is, the distance between two individuals represents the difference of minds and behavior between them.

Third, to make spiral collective motion of the whole group emerge, a periodic function (e.g., a sinusoidal function) is introduced into the interaction of individuals in our model. Inspired by the Lennard-Jones potential proposed in [32], the interaction function among individuals in our model is defined as follows.

$$f(x)=\left\{ \begin{array}{l} k_{1}(\frac{1}{x}-\frac{1}{d_{e}}),\;if\;d_{l}<x<d_{e}\\ k_{2}\sin[\frac{(x-d_{e})\pi }{d_{h}-d_{e}}],\;if\;d_{e}<x\leq \frac{d_{e}+d_{h}}{2}\\ k_{3},\;if\;x>\frac{d_{e}+d_{h}}{2}\\ 0,\;others \end{array}\right.$$ (1)

In Eq (1), the parameter *d*_*e*_ represents the expected distance. The parameters *d*_*l*_ and *d*_*h*_ represent the lower bound on the distance between two individuals that can generate repulsive interaction and the higher bound on the distance between two individuals that can generate attractive interaction, respectively. The bounds on attraction and repulsion in the traditional sense are inspired by the Lennard-Jones potential proposed in [32], and the effects of attraction and repulsion between two individuals have been used to simulate real interactions in a group of animals (e.g., birds or fishes). For instance, in [4], Couzin et al. show a self-organizing model based on this kind of interaction and use it to explain the spatial dynamics of an animal group.

In our paper, quite differently, we change the traditional meaning of the upper bound on the distance between two individuals that can generate attractive interaction; that is, the formula of attraction is changed into two parts. When the distance between two individuals exceeds a threshold value, which is set as (*d*_*e*_ + *d*_*h*_)/2, then the attraction will maintain the maximum value (we assume the value of parameter *k*_*3*_ is equal to the value of parameter *k*_*2*_). This idea is suitable for researching the topological interactive relationships among different individuals in a group. Different from the interactive relationship of metric distance, each individual can obtain the information of the *K* nearest individuals, regardless of how far away these neighbors are, and this parameter *K* is the number of topology. It is worth stressing that using this idea, we are able to construct a more intelligent model, and each individual is eager to obtain the information (e.g., decision process and behavior) of the *K* individuals that hold the most important information for it (or the easiest information to obtain since the easier the information is to obtain, the more eager the individual is to obtain it because of the similar way of thinking). It is possible for us to change the research object from animals to human beings. In our paper, we try to find a new point of view from which to analyze the interactions among a group of people and to explore the mechanism of interactions in human behavior.

In discussions about the interactions among a group of people, the concept of synergetics has appeared [33,34]. As described in [33], Haken put forward that the regularity found in the form of fog and the aggregation of cells is able to explain phenomena in sociology, namely, the behavior of all the people in a group seems to tend to a new idea (or fashion) and people in the group start to behave similarly after a short time. We are inspired by the synergy mechanism in various kinds of systems mentioned in [33] and [34], so we try to use the method of topological interaction, which has been successfully used to describe collective behavior in the world of animals, to explain phenomena in human behavior.

**Fig 1:**
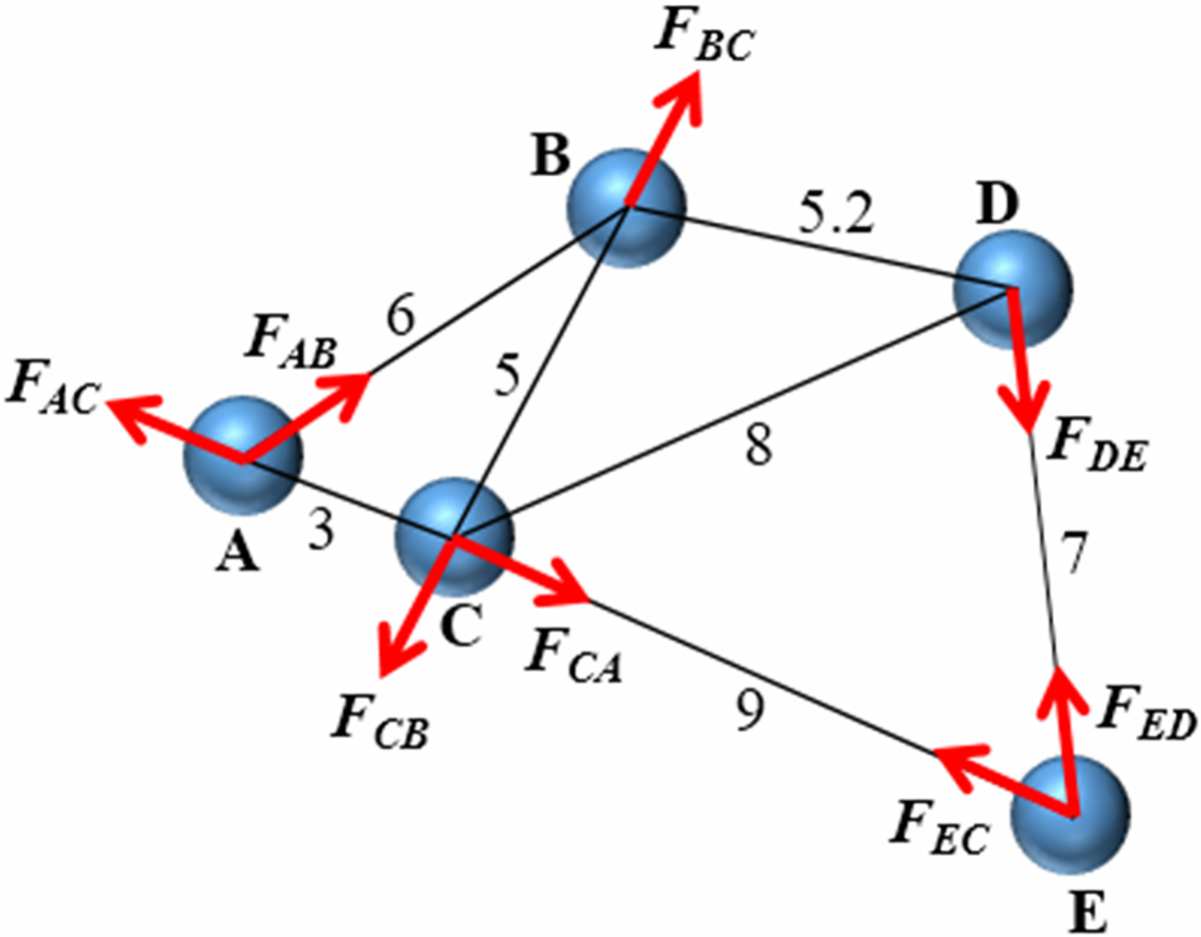
A simple example of interactions between individuals based on the rules in Eq (1). (*N* = 5, *K* = 2, *d*_*l*_ = 0, *d*_*e*_ = 5.2, *d*_*h*_ = 12, (*d*_*e*_ + *d*_*h*_)/2 = 8.6).

In our model, the problem of force and how the group is able to move will be discussed in more detail as follows. First, we give a simple example to illustrate the forces between individuals. As shown in Fig 1, every individual is acted upon by attraction and repulsion from the nearest *K* individuals, and the magnitude of the resultant force between a pair of individuals is based on the distance between the two individuals (see Eq (1)).

Second, the formula for the force is shown in Eq (2), which indicates how the group starts to move and keeps moving. For any individual *i* (*i* = 1, 2, …, *N*), the magnitude of the resultant force is based on the difference between the attraction and repulsion. The parameters *a* and *b* are the different weights of attraction and repulsion, respectively. The magnitude of attraction (repulsion) on individual *i* is the resultant force of attraction (repulsion) from the other individual *j* (*j* = 1, 2, …, *N*, *j* ≠ *i*). In three-dimensional space, the formulas shown in Eq (2) and Eq (3) need to be calculated for the coordinate components on the three axes. Third, the resultant force is the driving force that makes all individuals in the group move. In Eq (3), we make the following simple assumptions. The direction of the velocity is in accordance with the direction of the resultant force, and the displacement variation is proportional to the magnitude of the resultant force. With these assumptions, we can construct a meaningful collective motion phenomenon based on a series of relatively simple rules.

$$F_{i}\left(t\right)=aF_{i}{}^{a}\left(t\right)-bF_{i}{}^{r}\left(t\right)=\sum_{j=1}^{N}aF_{ji}{}^{a}\left(t\right)-bF_{ji}{}^{r}\left(t\right)$$ (2)

$$S_{i}\left(t+1\right)-S_{i}\left(t\right)=cF_{i}\left(t\right)$$ (3)

## Results

### The explanation of simulation methods

In this section, many simulations are performed to discover different kinds of collective motions, which are based on interactions among individuals in the group under different initial conditions. In the simulations, the number of individuals is from 1 to 50 and the number of topology is from 0 to 49. According to the simulation results, we find that the initial locations play a significant role in affecting which types of collective motions will emerge from the whole group, rather than whether collective motions will emerge. Therefore, we select two groups of characteristic initial locations of individuals to research the emergence of group behavior. One circumstance is an apparently isolated individual within a group that consists of loosely distributed individuals, and the other circumstance is that collective behavior has already emerged among some individuals in the group in the initial state. Other parameter values are selected to guarantee the stability of the model. We use MATLAB to simulate the model and gather data from the results. Then, we organize the data about the network properties and use R to perform statistical analysis. According to Eqs (1–3), we start the simulation with a group of representative initialization parameters, which are listed in Table 1.

**Table 1:** Parameter values in the simulations.

Parameter	The meaning of the parameter	Parameter value
*d*_*l*_	Lower bound on the distance that can generate repulsion	0
*d*_*e*_	Expected distance	5.2
*d*_*h*_	Higher bound on the distance that can generate attraction	12
*k*_1_	Coefficient	1
*k*_2_	Coefficient	0.3
*k*_3_	Coefficient	0.3
*a*	Weight of attraction	0.08
*b*	Weight of repulsion	0.2
*c*	The proportional relationship between displacement variation and resultant force	1
*t*	Simulation steps	20000
*N*	Number of individuals	1~50
*K*	Number of topology	0~49

### Various collective motions

**Fig 2:**
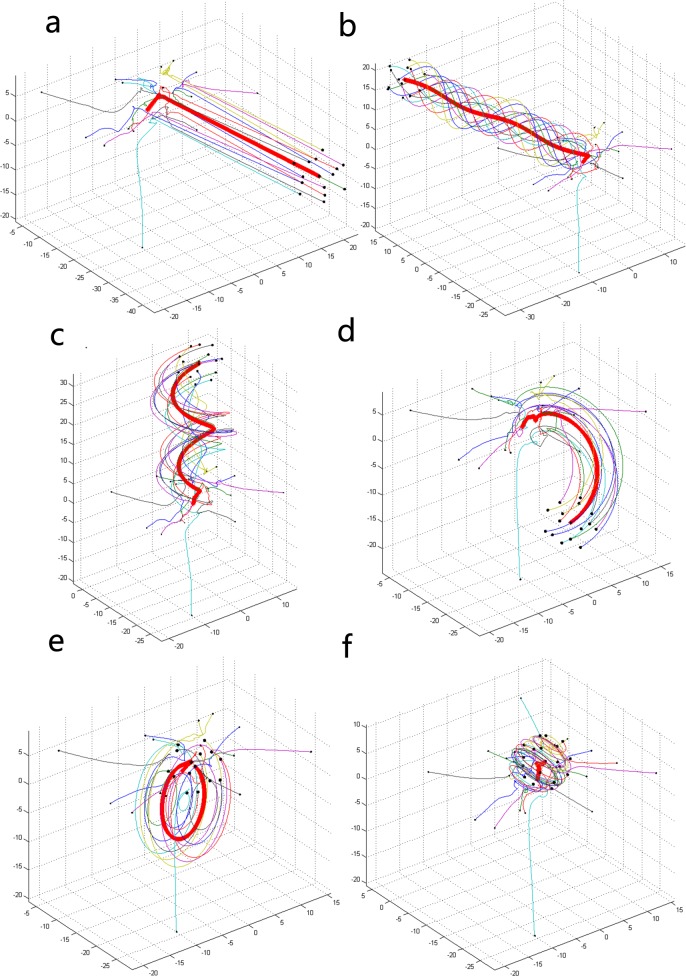
Various collective motions under different combinations of *N* and *K*. ((a) *N* = 15, *K* = 7; (b) *N* = 15, *K* = 8; (c) *N* = 16, *K* = 7; (d) *N* = 16, *K* = 10; (e) *N* = 15, *K* = 9; (f) *N* = 20, *K* = 11).

**Fig 3:**
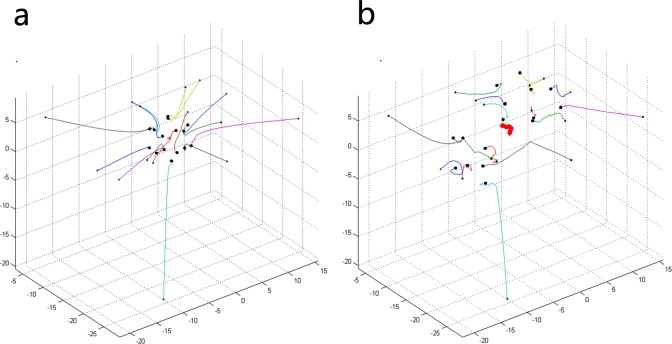
Special collective motions under different combinations of *N* and *K*. ((a) *N* = 15, *K* = 14; (b) *N* = 16, *K* = 4).

As the size of the group (*N*) and the internal topological interactive relationships (*K*) change, various types of collective behavior of the group emerge. As shown in Fig 2, we consider six types of successful collective motions. It is worth noting that the red thick line shows the average position of the whole group, which also intuitively represents the tendency of the collective motion. The initial positions of individuals are set as a group of constant data in the process of simulation, and we can explore the relationship between the various types of collective motion and the combinations of *N* and *K*. Then, we will analyze these types of collective motion in detail. To analyze the outcomes conveniently, we mark the collective motion in Fig 2A as type 1. Similarly, the collective motions from Fig 2B to Fig 2F are marked as types 2~6, and types 7 and 8 are shown in Fig 3A and 3B. Intuitively, individuals participating in type 1 or 2 motion will eventually go straight along a certain direction. However, individuals participating in type 5 or 6 motion orbit a central point, and the whole group moves like a torus. Types 3 and 4 seem to be combinations of types 1 and 5. Individuals travel along an approximately certain direction and orbit a central line when they move. Type 7 motion seems to be a special type of collective motion because all individuals aggregate quickly and the whole system tends to a stable state immediately. Moreover, type 7 motion is often found when the value of *K* is large (especially when *K* = *N*-1). Type 8 motion is just unformed collective motion, and it is often found when the value of *K* is small (especially when *K*<5).

We find two important indicators in the simulations: straightness and helicity, and the size relationships among the types of collective motion can be summarized as follows.

$$S_{1}>S_{2}>S_{3}>S_{4}>S_{5}\geq S_{6}=S_{7}=0$$ (4)

$$H_{6}\geq H_{5}>H_{3}>H_{4}>H_{2}>H_{1}=H_{7}=0$$ (5)

Different from the four collective dynamical behaviors used to investigate the spatial dynamics of an animal group in [4], in our model, we have more kinds of collective behaviors and a special interactive function, which is given as Eq (1). The attraction attains the maximum value when the distance between two individuals exceeds the threshold (*d*_*e*_+*d*_*h*_)/2. This concept is not easy to explain in the animal world. Nevertheless, from the point of view of human beings, one person will make every effort to obtain the information or viewpoint from another person if he deems it important and valuable to him. There are several characteristics of the interaction between two people (e.g., consciousness). For instance, it is impossible that the consciousness (or viewpoint) of one person is absolutely the same as that of another person, and it is inevitable that individuals will influence each other in the process of interaction (e.g., communication). When two people reach a consensus (relatively similar viewpoints between them) after the process of interaction, a stable state in which the individuals maintain the expected distance from each other and travel along the same direction will eventually emerge, as shown in Fig 2.

From the point of view of interactions among people, it seems more suitable to regard straightness as the advancement and development of a group (represented by *A*) and to regard helicity as the behavior of the game among people (represented by *G*). Therefore, we can analyze the collective motions in Fig 2 and Fig 3 from a new perspective. Type 1 represents a highly consistent group. People in this group quickly reach a consensus after a short interaction process and move straight toward the goal that can achieve the most benefit for all individuals in the group. This group will maintain a stable state when there is no external disturbance. Type 2 is similar to type 1. However, compared with type 1, type 2 has a lower degree of *A* and a higher degree of *G*, namely, there exists a slight divergence in this group, but this has no effect on the ability of the whole group to pursue the maximization of benefits by way of collaboration. The degree of *G* is the highest in type 5 and type 6. People in the group are likely to maintain the state of the game, and they will not reach a consensus because none of them can persuade the others. This phenomenon that every individual sticks to his or her own opinions results in the whole group never getting ahead and just going in circles. This state may be described as involving a continuous debate among people, and the group will not achieve a win-win situation. This kind of state is not what we want to see because there is no advancement and development. In type 3 and type 4, there is a balance between the degrees of *A* and *G*. Type 3 is the spiral collective motion mentioned above. It widely exists in real life, and this kind of state may be regarded as the expected state of development in collaboration and competition. It also reflects the law of development, which is significant for us to explore the mysteries in our lives. Type 7 is a special collective motion that emerges when the value of *K* is very large (especially when it is close to *N*). Everyone is able to obtain enough information from others, and the whole group quickly reaches a perfectly stable state because the information in this circumstance is open and highly transparent. Everyone can achieve the maximum satisfaction and will not want to change their behavior. Hence, the degrees of *A* and *G* are both equal to 0. Although this kind of ideal circumstance may not exist in real life, it may emerge under some additional conditions (e.g., from a partial perspective). That is, the collective motions in Fig 2 and Fig 3 are all valuable for us to discover the rules of interactions among people.

### Fission behavior

**Fig 4:**
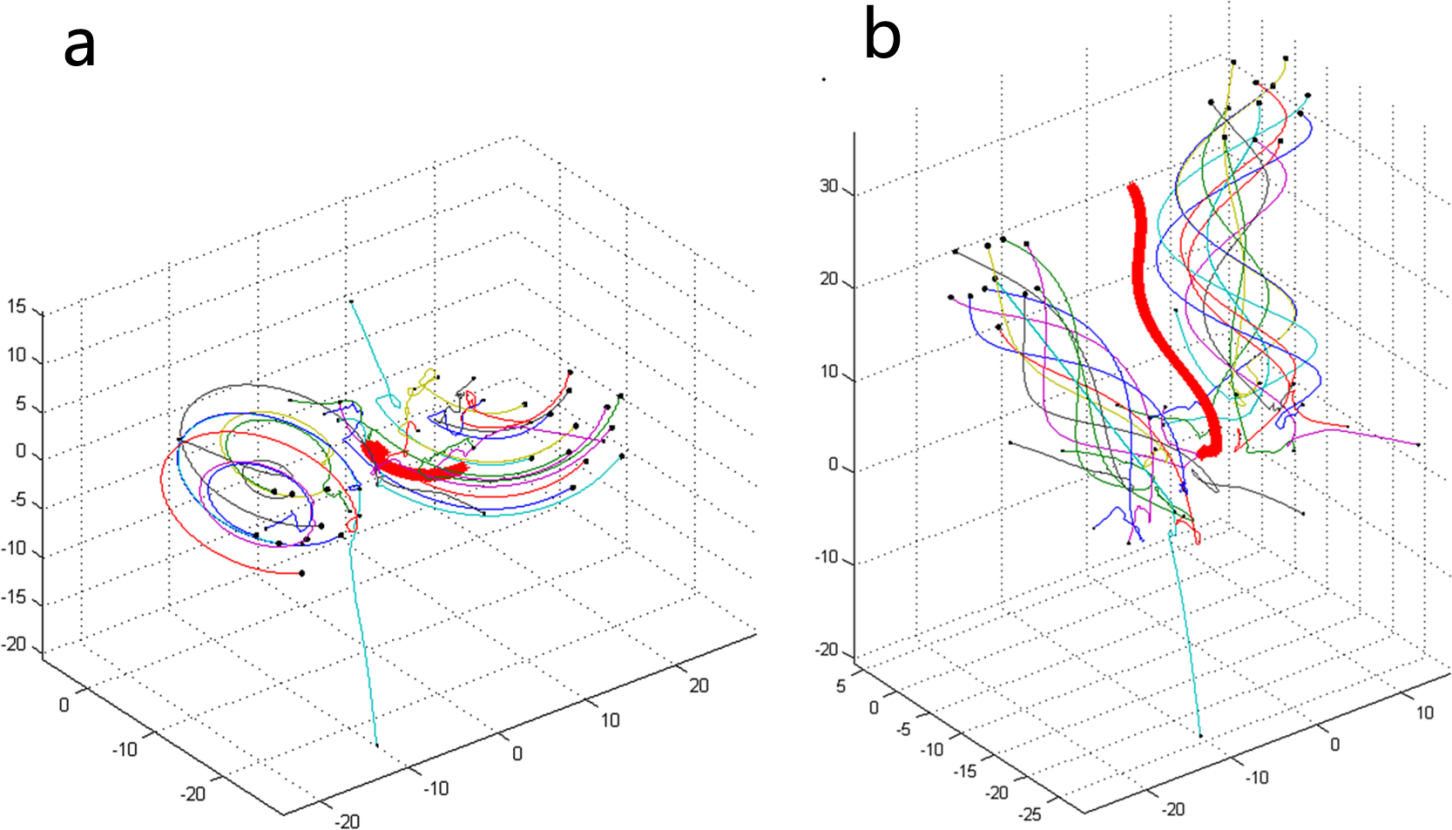
Two kinds of fission behavior under different combinations of *N* and *K*. ((a) *N* = 22, *K* = 5; (b) *N* = 23, *K* = 6).

In this section, another special collective motion will be discussed: fission behavior of a swarm system. Different from the collective motions in Fig 2 and Fig 3, individuals do not tend to gather into a single group. We find two types of fission behavior in our model. As shown in Fig 4, the difference between the two types lies in whether the types of the subgroups are the same or not. It is worth noting that the types of subgroups are the same as the types of collective motion mentioned in the previous section.

**Fig 5:**
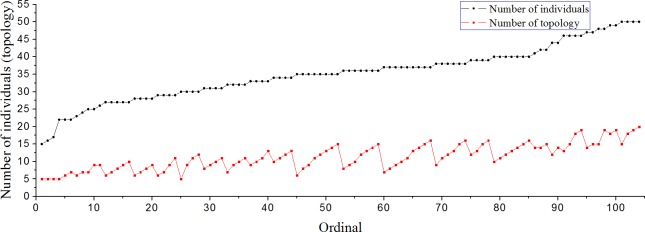
The tendencies of combinations of *N* and *K* that can generate fission behavior. (1 ≤ *N* ≤ 50).

In Fig 5, as the number of individuals increases, the value of *K* in the combination of *N* and *K* also increases, which can make the fission behavior of a group emerge. However, it never exceeds *N*/2 because in a topological interactive relationship, only the nearest *K* individuals are considered, and it can also be found that the number of subgroups does not exceed *N*/*K*. Thus, from the point of view of the phenomenon that the group separates into two subgroups, the requirement that can lead to the emergence of fission behavior is *K* ≤ *N*/2; this is confirmed in Fig 5. In addition, the minimum value of *K* for fission behavior is 5, and this is the same minimum value of *K* under which the group can generate collective motion. Thus, fission behavior can be recognized as a special type of collective behavior. However, in our model, there are at most two subgroups in fission behavior. This may be related to there being fewer individuals in the simulations, but it does not affect the analysis of the rules of interactions among people.

From the point of view of people, fission behavior represents the emergence of a conflict of opinion in the interactions among individuals in the group. It is different from type 5 and type 6, in which individuals in the group maintain the behavior of the game at all times and there is no advancement and development of the whole group. As shown in Fig 4, some or all individuals in the group escape from the state of the game. Two new subgroups reach their own consensuses and forge ahead in new directions to pursue the maximization of benefits. Furthermore, as the value of *K* increases under a constant *N*, collective motion appears for the first time within the range of threshold values (five to seven), and fission behavior may emerge when *K*∈[5,*N*/2]. Apart from the combinations of *N* and *K* that can generate fission behavior, the rest of the combinations of *N* and *K* (here, the value of *K* should not be less than that when collective motion appears for the first time) can all lead to the emergence of the collective motions shown in Fig 2 and Fig 3. Hence, from the point of view of the number of topology *K*, we are able to analyze whether fission behavior can emerge from a group of people and the relationships between fission behavior and collective behavior. In addition, the value of *K*/*N* may represent the degree of transparency of valuable information in the whole group system. From the data in Fig 5, we discover that the value of *K*/*N* lies within [16.7%, 43.2%] under the circumstances of fission behavior, and the average of those values of *K*/*N* is 31.8%. Here, the value is shown as a percentage because we realize it may be suitable to describe it as the percentage of the degree of the disclosure of valuable information in the whole group system. Moreover, it is also meaningful to discover the transition rules between fission behavior and collective behavior, and these conclusions may contribute to helping us find the rules in our real lives in the future.

### Various initial locations

**Fig 6:**
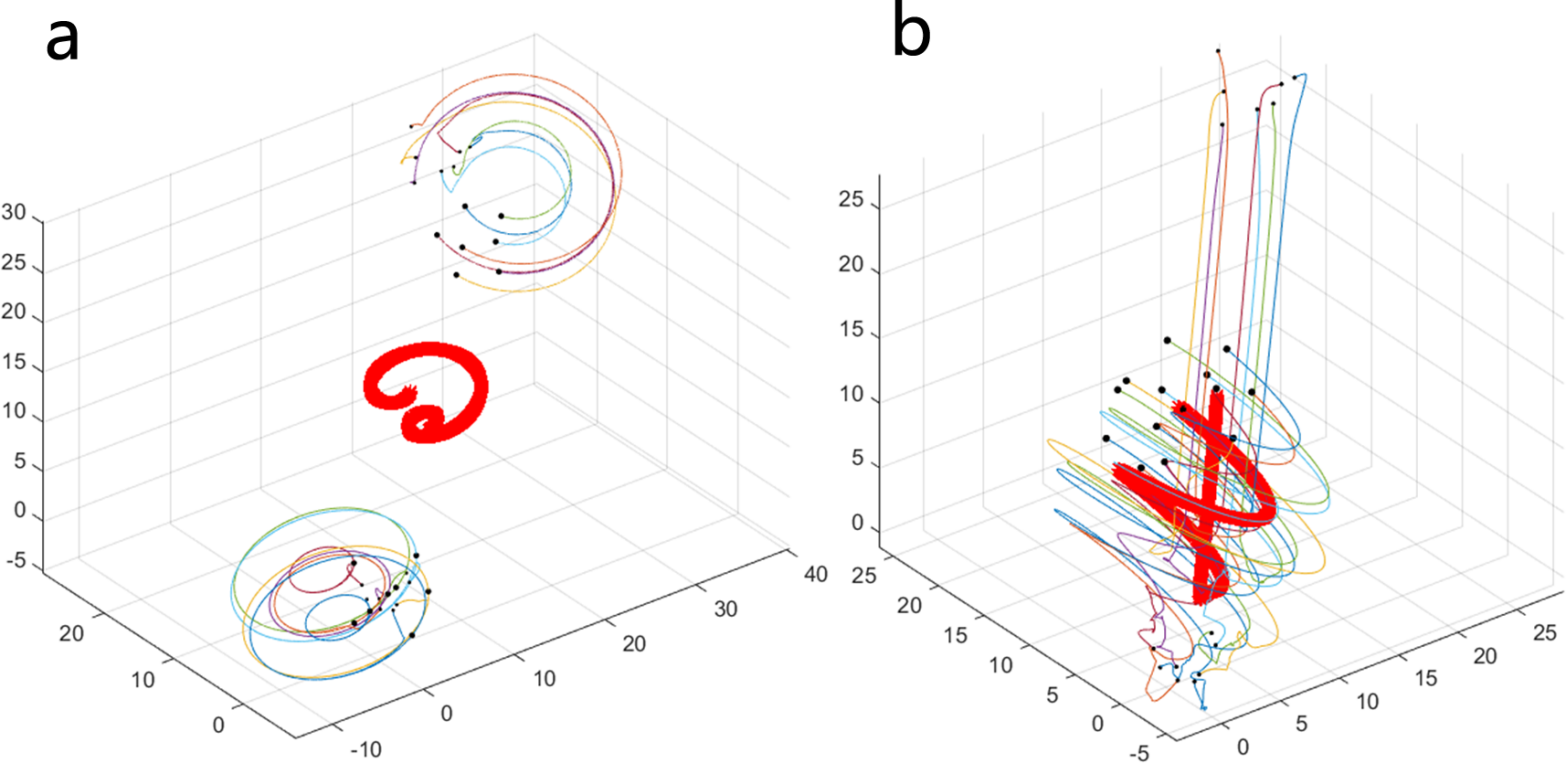
Two subgroups at initial locations under different combinations of *N* and *K*. ((a) *N* = 15, *K* = 5; (b) *N* = 15, *K* = 7).

**Fig 7:**
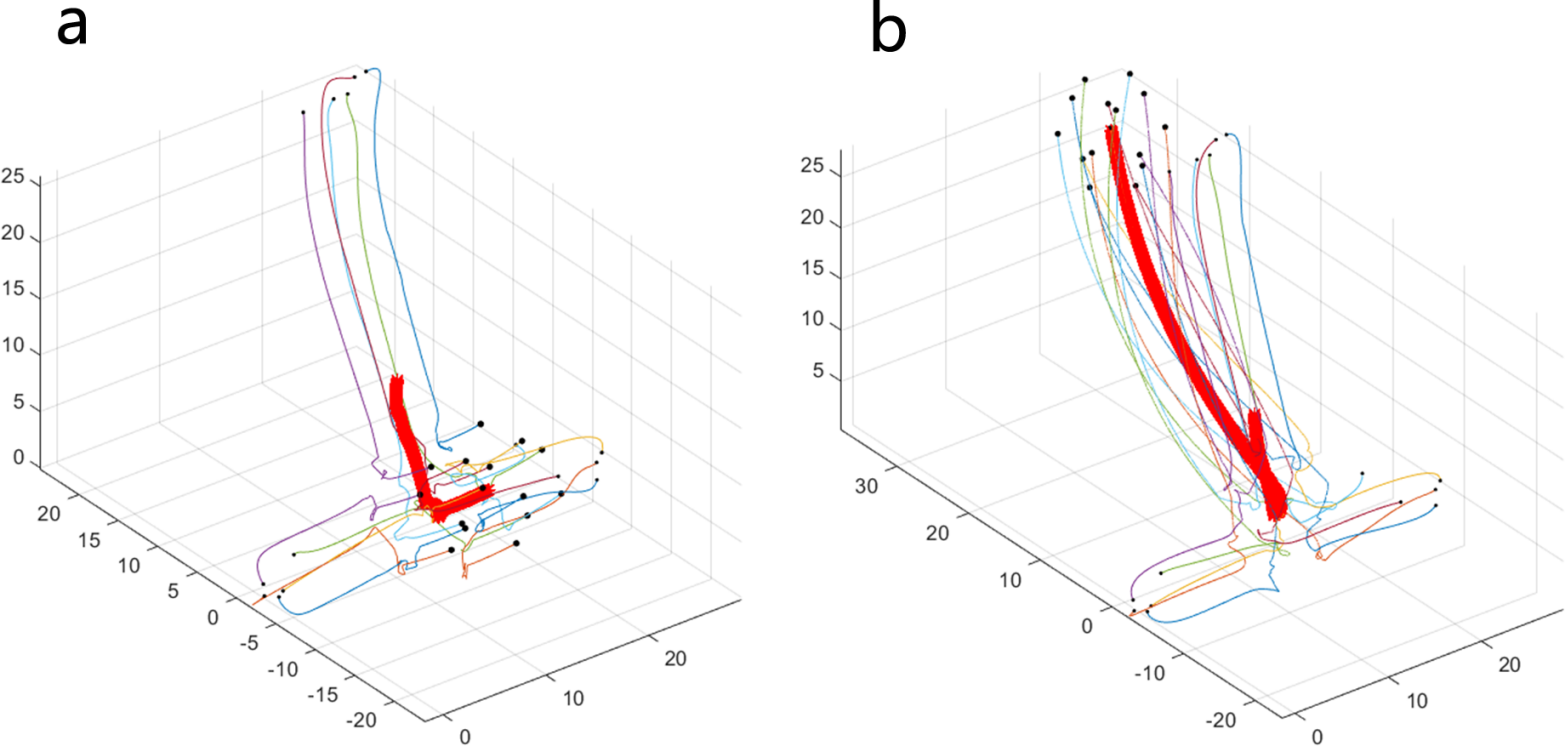
Three subgroups at initial locations under different combinations of *N* and *K*. ((a) *N* = 15, *K* = 5; (b) *N* = 15, *K* = 7).

In the original simulations described above, the initial locations of 50 individuals are well distributed. Here, some special circumstances of initial locations will be discussed. As shown in Fig 6, there is a distance between the two subgroups in the initial state. When the number of topology *K* is equal to 5, the two subgroups maintain the initial state of separation and reach their own stable states. However, when the value of *K* increases, collective behavior emerges from the two subgroups, and they eventually move together as a whole group. The reason for this phenomenon is that the possibility of the emergence of collective behavior from individuals in the system increases as the value of *K* increases, and the phenomenon of the change from Fig 6A to Fig 6B can be described as a transition process from quantitative changes to qualitative changes. A circumstance that contains three subgroups in the initial state is shown in Fig 7. Different from Fig 6, when the value of *K* is equal to 5, the collective behavior has already emerged. At first, the three subgroups move in the direction of the center point of their locations. Then, they gather successfully and move together in the same new direction. In this section, we mainly want to show some additional results about the role of initial conditions, and it is meaningful to consider the problem of whether and how some kinds of collective motions emerge from the subgroups in the initial state.

### Analysis of network properties

**Fig 8:**
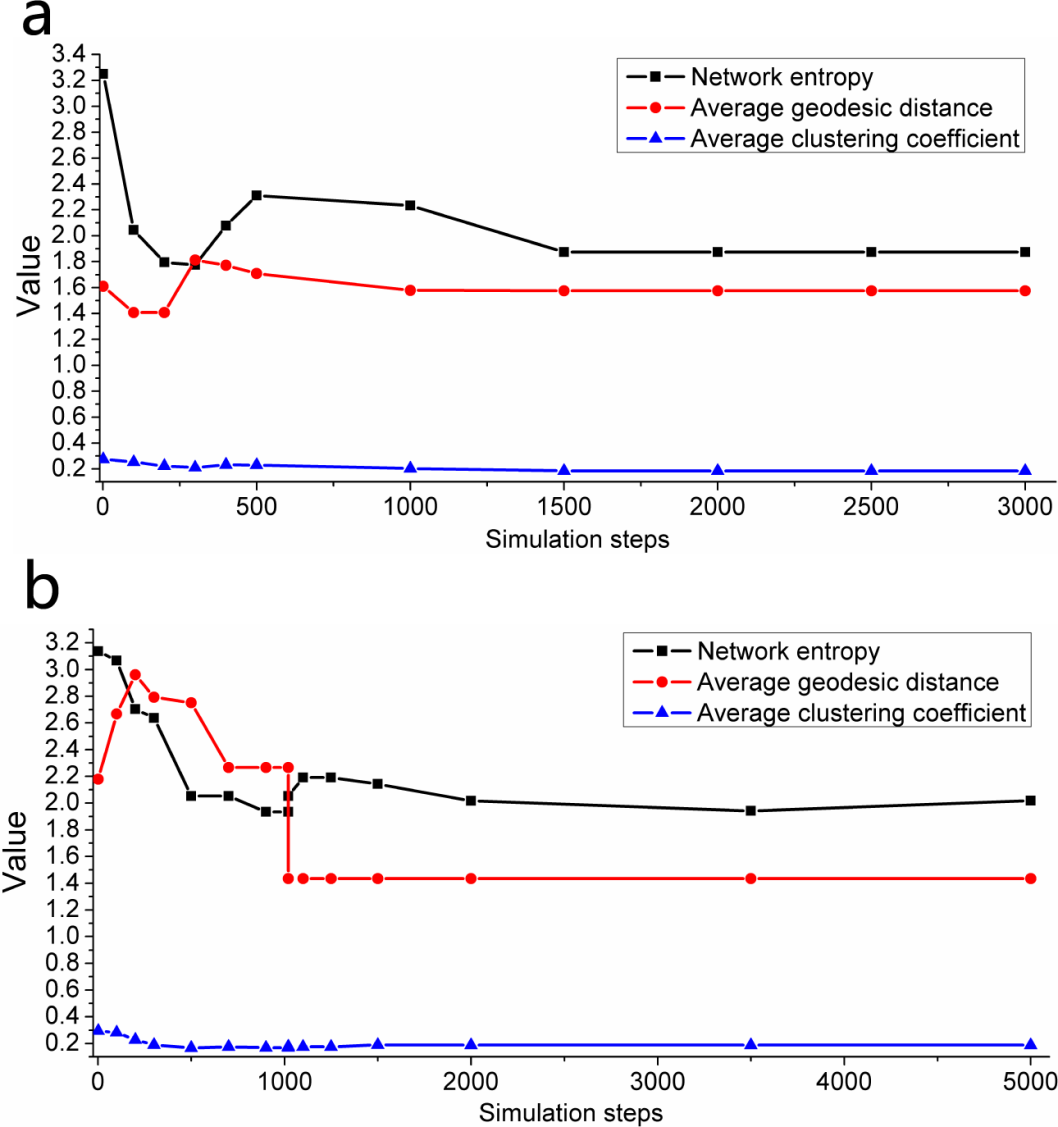
The quantitative variation of various network properties under different combinations of *N* and *K*. ((a) *N* = 16, *K* = 7; (b) *N* = 23, *K* = 6).

In this section, we attempt to explain the model from the perspective of network features because topological interaction relationships are suitable for analysis using the theory of complex networks. First, many network properties (e.g., network entropy, average geodesic distance, average clustering coefficient and network density) are chosen to find rules in the analysis procedure. Since the motions in our model can be divided into two kinds, we select the spiral collective motion in Fig 2C as a representative case of collective motions and the fission behavior in Fig 4B as a representative case of fission motions. In Fig 8, every property eventually tends to a constant value, which means that the whole group gradually tends to a stable state in the process of interactions among individuals. It is worth noting that a critical point of fission behavior can be found in Fig 8B. After the critical point of fission behavior, the whole group splits into two subgroups. The value of network entropy apparently increases, and the average value of the geodesic distance in every subgroup decreases, which shows the close relationships among individuals within the subgroups. Here, the average value of the geodesic distance shown in Fig 8B is calculated from the geodesic distances that still exist between individuals. That is, the connections between individuals that belong to different subgroups disappear, and the value of the geodesic distance between them is infinity. Moreover, the trajectories of network entropy and average geodesic distance all apparently go through a process that tends to an equilibrium point before the critical point of fission behavior; then, after a distinct jump at the critical point of fission behavior, they reach a new equilibrium state that is different from the former equilibrium state. This phenomenon is in accordance with the ideas in [33]; to be specific, Haken put forward that there is an internal automatic mechanism that affects the whole system’s change from chaotic to ordered or from one ordered state to another ordered state in the fields of economics, sociology and politics. Thus, it is meaningful to determine the mechanism in the above collective motions, which may help us learn more about the behavior of human beings.

**Fig 9:**
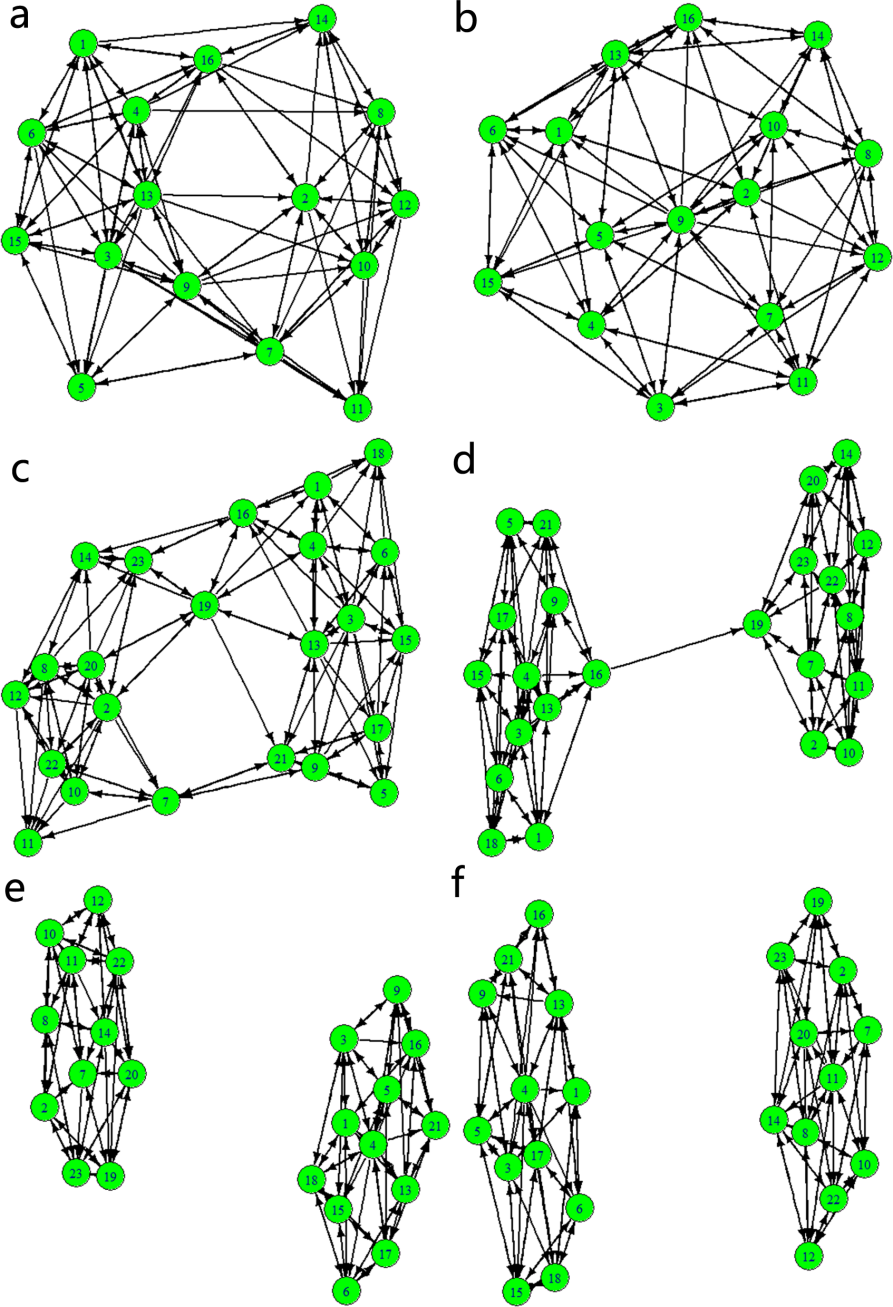
Network graphs for different steps and different combinations of *N* and *K*. ((a) *N* = 16, *K* = 7, step = 1; (b) *N* = 16, *K* = 7, step = 3000; (c) *N* = 23, *K* = 6, step = 1; (d) *N* = 23, *K* = 6, step = 1020; (e) *N* = 23, *K* = 6, step = 1021; (f) *N* = 23, *K* = 6, step = 5000).

**Fig 10:**
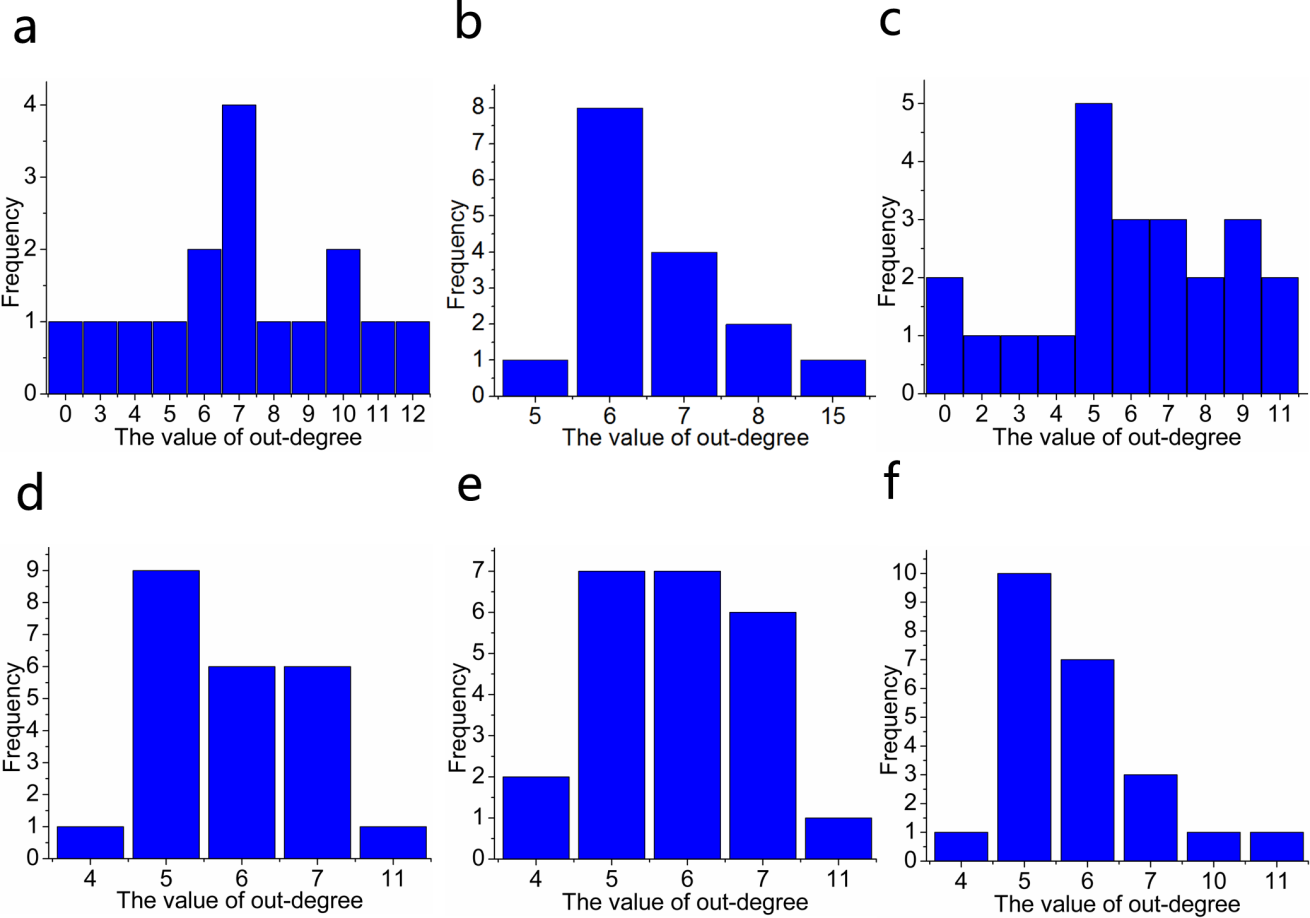
Distribution of the out-degrees of individuals for different steps and different combinations of *N* and *K*. ((a) *N* = 16, *K* = 7, step = 1; (b) *N* = 16, *K* = 7, step = 3000; (c) *N* = 23, *K* = 6, step = 1; (d) *N* = 23, *K* = 6, step = 1020; (e) *N* = 23, *K* = 6, step = 1021; (f) *N* = 23, *K* = 6, step = 5000).

Second, to intuitively show the relationships of interactions among individuals in the two cases in Fig 8, the network graphs that correspond to those two cases are shown in Fig 9. In addition, we discover some rules in the distributions of the in-degrees and out-degrees of the individuals, which are shown in Fig 10. To be specific, the in-degree of every individual is the same, and it equals the number of topology *K* in the evolutionary process. In addition, when the whole group is in a stable state, almost all the out-degrees of individuals distribute around the number of topology *K*, except that the out-degree of a single individual is obviously larger than (approximately double) those of the others. In Fig 9, a visualized evolutionary process of fission behavior is shown, which also contains the details of the critical point of fission behavior. According to Fig 9 and Fig 10, the whole group tends to an equilibrium point before the critical point of fission behavior since the distribution of the out-degrees of individuals satisfies the rules found in the stable state. However, after the critical point of fission behavior, the whole group breaks up into two subgroups. Eventually, the subgroups all tend to their own stable states. This phenomenon conforms with the conclusions of Haken mentioned above. Nevertheless, the reason for the conclusions mentioned above is worth analyzing and discussing. We propose that the phenomenon of the distributions of the in-degrees and out-degrees of individuals may be explained as follows. The in-degree of an individual can be viewed as the number of neighbors that have an interactive relationship with the individual, that is, the number of nearest neighbors from which the individual can obtain information. Therefore, it conforms with the concept of the number of topology and is always equal to the number of topology. The out-degree of an individual represents its ability to influence the viewpoints or behavior of others. This ability may be reflected as authority, prestige and treasure. It is common sense that collective behavior more easily emerges from individuals in the group if there is a leader in the group. Otherwise, the group is likely to fall into a state of disorder and chaos. Although the group is in the state of disorder and chaos at first, a new leader may appear during the evolutionary process. This also causes the group to tend to a stable state, and the reason may be a common goal that all individuals in the group expect to achieve.

**Fig 11:**
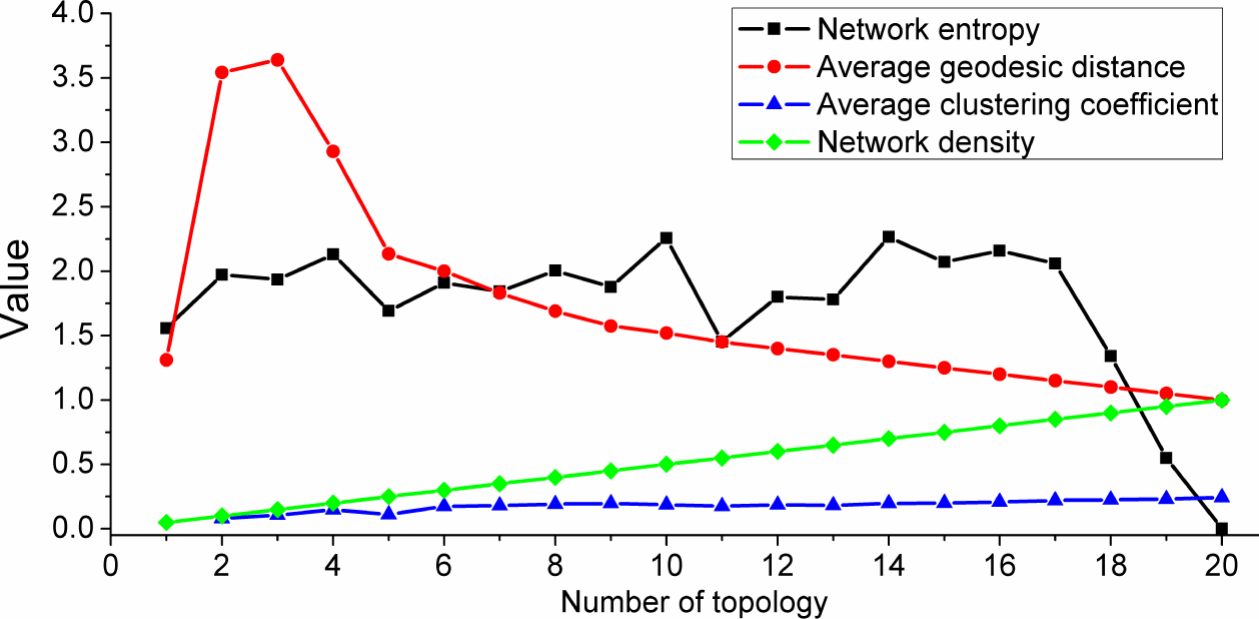
The quantitative variations of various network properties under different *K* and constant *N*. (*N* = 21, 1 ≤ *K* ≤ 20).

Third, we seek to understand the relationships between the number of topology and many properties of the network. As shown in Fig 11, the number of individuals *N* is a constant, and the following conclusions can be drawn.

$$D=\frac{K}{N-1}$$ (6)

$$g\approx 2-\frac{K}{N-1}$$ (7)

The network density *D* is completely determined by the values of *N* and *K*, and it remains constant during the evolutionary process. However, other properties change over time. The data shown in Fig 11 all correspond stable states under various circumstances. A special case occurs when the number of topology *K* is equal to 1, which causes the network graph to be unconnected. This also causes the data of average geodesic distance and network density to seem different from the data under other circumstances. In addition, the average geodesic distance *g* is approximately inversely proportional to the number of topology *K* when *K* is greater than or equal to 5; the reason is that 5 is the threshold for the emergence of collective behavior, which also makes the average geodesic distance change regularly. The average clustering coefficient roughly increases with the increase of the value of *K*, and this implies that better aggregation influence is consistent with a higher degree of topological connection. The value of the network entropy under the circumstance of type 7 shown in Fig 3A is obviously less than those of other types. In particular, when the value of *K* is equal to *N*-1, the value of the network entropy under the circumstance of type 7 is 0. This implies that the level of order of type 7 is higher than those of other types, and the circumstance of type 7 also has the highest value of network density. When *K* is equal to *N*-1, the network is just a *k*-regularity graph because every individual has the same in-degree and out-degree, and the value of the network entropy is 0. These features conform with the characteristic of *k*-regularity graphs. Moreover, we speculate that other types of collective motions may conform with the characteristics of the small-world network. The reason is that collective motions are generated when *K* exceeds the threshold of the emergence of collective behavior, which implies a relatively higher level of determinacy. In addition, apart from type 7, the collective motions are distributed randomly, which indicates a relatively lower level of randomness. Thus, we draw the conclusion stated above, which certainly needs to be confirmed in future work.

## Discussion

In this paper, we discover that the outcomes of our model may be strongly associated with the viewpoints presented in the papers of many scholars. For instance, in [22], Ballerini et al. discovered that compared with the interactive relationship of metric distance, topological interaction shows some superior characteristics, such as robust cohesion and higher biological fitness. In addition, they found that the interaction mechanism is shaped by evolution to ensure the cohesion of individuals in a whole group, regardless of how complex the natural environment is, and various shapes of aggregations and types of splitting can emerge via interaction with a fixed number of neighbors (6 to 7). However, if we consider the interactions among human beings, it is doubtful that people are always interacting with a fixed number of neighbors because humans and human society are more complex than animals and animal world. Although the exact number of neighbors that people interact with has not been confirmed, we can simulate all the values of *K* to determine the underlying mechanism suitable for explaining the collective behavior of human beings. As has been analyzed in previous sections, the value of *K* can represent the degree of topological interaction in the whole system and how much information each individual can obtain from neighbors. It also reflects the transparency of valuable information in the system composed of *N* individuals, which can represent a local environment rather than the whole environment because the process of self-organization in our model focuses on the relationships of interactions among *N* individuals and ignores the possible exchange of information with the outside environment. As shown in the results section, the aggregation of the whole group is gradually enhanced as the value of *K* increases, and a highly aggregated process emerges when *K* reaches the maximum value. These phenomena may fit the human behavior under different degrees of transparency of information in the environment. The degree of transparency determines the amount of information one person can obtain, and the change in the direction and style of one person’s behavior is based on the obtained information. However, we suggest that the number of neighbors that people are always interacting with may be within [5, *N*/2] because the phenomena of collective and fission behaviors, which widely exist in our lives, can emerge when the value of the number of topology *K* is within this range.

The value of *K* also determines whether the system will tend to a stable state because the stable state of the system is the necessary prerequisite for the emergence of collective motion. Furthermore, in [23], Young et al. found that the robustness of the system is related to the value of *K*, and in their paper, several conclusions were drawn. For instance, the graph of topological relationships in the system remained disconnected for *K* equal to 1 and 2 but was almost connected when *K* = 5, and the average robustness per neighbor reached the peak value at *K* = 6 or *K* = 7. It is interesting to note that some similar conclusions are obtained in our model. For example, obvious collective motion will not emerge from individuals in the system when *K*<5, and various collective motions begin to emerge when *K* = 5 (the minimum value of *K* at which collective behavior emerges in the system). After many groups of simulations, we discover that collective behavior emerges from the system for the first time when *K* belongs to five to seven, and this range of values has been called a threshold of the emergence of collective behavior.

A famous experiment on the small-world effect was carried out in [35]. In this paper, it was found that the average number of intermediaries needed to pass a letter from a starter to a target is 5.2, and any two people are separated by approximately six people on average, which is called six degrees of separation. It is interesting to find that this value is in the range of the threshold of the emergence of collective behavior in our model, and we suggest that there may be a connection between them from the viewpoint of complex networks. In [24], Komareji & Bouffanais found that every agent interacts on average with a fixed number of neighbors, irrespective of the distance between them, and the number of neighbors *K* changes with the group size *N*. The group will remain connected at all times if the value of *K* is at least approximately 6 or 7, which is in accordance with the conclusions in [22]. Interestingly, in [24], they also discovered that 4 to 5 interacting neighbors are necessary to ensure the group’s connectedness and effectiveness. The dynamic swarm signaling network (SSN) is a homogeneous and clustered small-world network if connected. The small-world effect can be used to explain many phenomena, such as the response of a biological swarm to an external stimulus. In [36], Dodds et al. performed an experiment on forwarding messages to acquaintances, and they discovered that social searches can reach their targets in a median of five to seven steps. It is amazing that this range is equal to the threshold of the emergence of collective behavior, which suggests that there may be a relationship between them.

Most group behaviors in the societies of animals and human beings are based on cooperation. In [37], Nowak put forward five rules for the evolution of cooperation and described cooperation as the decisive organizing principle of human society, which contains complex games of cooperation and defection. People frequently fall into the social dilemma, which is a special situation in which individual rationality is at odds with group outcomes. If an individual prefers to pursue maximum collective interests, then he will choose cooperation. Otherwise, he will choose defection, as personal interests are more important to him. As shown in the results section, the helicity can measure the game behavior among people. Compared to straight-line motions, spiral motions likely represent a state of stagnant development and spinning in the same place. This conforms with the idea that people always hesitate to choose a possibly better strategy under the state of the game. Generally, helicity is almost the exact opposite of straightness. Thus, the degree of helicity will increase as the degree of straightness decreases. The possibility that individuals will choose defection increases as the degree of game behavior increases, and this also leads to a decrease in focus on collective interests. Likewise, the possibility that individuals will choose cooperation increases as the degree of game behavior decreases, and this leads to increased focus on collective interests. Similarly, conclusions on the straightness, which is regarded as a measure of the advancement and development of a group, are the opposite of those on the helicity mentioned above. In addition, we are curious about network reciprocity and group selection, which were mentioned in [37]. Nowak noted that in spatial structures or social networks in real populations, some individuals interact more often than others, and cooperators can prevail by forming network clusters where individuals are able to help one another. Beyond that, Nowak also elaborated that a group can split into two when it reaches a certain size, and groups of cooperators have a higher rate of splitting into two. Under the mechanism of group selection, groups of cooperators seemed to be more successful than groups of defectors, and groups of cooperators were less likely to go extinct. Our model may support the conclusion mentioned above. More precisely, the number of circumstances of fission motions is apparently less than that of collective motions. An explanation of the fission motions in our model is that individuals in the group discover that they cannot realize the expected collective interests if they choose cooperation and cannot realize their expected personal interests if they choose defection. Under the effect of feelings that people do not satisfy the status quo, individuals in the group will pursue their own expected interests and rearrange into new groups. After that, these individuals will continue to pursue the expected interests of the new groups, which may be collective interests or personal interests.

As collective behaviors widely exist in various kinds of systems, we find that our results are connected with these phenomena. For example, the collective behaviors of a mass of neurons in the nervous system are complex because of the intrinsic and external uncertainty, and a kind of spatial distribution called a spiral wave could be developed in the network, which is connected with the regular nearest-neighbor type [5]. It is interesting to find that a special type of collective behavior called spiral collective behavior appeared in our results. A neuron model effective in reproducing many properties of electric activities of neurons was introduced in [8], and the authors discovered that appropriate networks could be designed to research the collective behaviors of neuronal networks. In addition, nonlinear and chaotic characteristics have been found in the collective behaviors of neurons [5,8]. These characteristics also emerge as the system tends to the stable state in our model, and we analyze the network properties of the model to research the intrinsic mechanism in the process of the emergence of collective behaviors. These similar characteristics found in the analysis of collective behavior also imply the effectiveness of our model in explaining these phenomena.

Apart from the collective behaviors in the nervous system, it is well known that there are various kinds of collective behaviors in the human world. Can the model describe the interactions among people? In the process of solving this problem, we found that our ideas have strong connections to many papers that research the interactions among people. For instance, in [9], Moussaïd et al. found that the dynamics of human crowd behavior are determined by two conditions: physical constraints induced by other pedestrians and social interactions such as communications among individuals. These two conditions conform with the interactive rules in our model. The interaction function among individuals in Eq (1) is a physical constraint, and the topological interactions among individuals (the rule of nearest neighbors) represents a way to communicate with other individuals. In addition, similar to the expected distance in our model, they defined the distance as a comfortable walking position that supports communication with other people. Different behaviors of human crowds also emerge based on those two conditions. A leader effect in pedestrian groups enables pedestrians who talk more to end up in the middle of the group, while listeners walk on the sides, and large groups usually split up into subgroups around those who talk most [9]. It is interesting that the leader effect also emerges in our model; that is, the distribution of out-degrees in the stable state implies that the out-degree of a leader is larger than those of other individuals. The value of out-degree represents the ability to influence other people. After the group splits up into two subgroups, a new leader will emerge in every subgroup and be surrounded by other individuals in the subgroup. In addition, in [10], Vorobeychik et al. performed some experiments in which individuals were allowed to communicate either with their immediate neighbors (locally) or with the whole network (globally). They discovered that global communication is more informative and efficient than local communication in improving coordination performance. In our model, the circumstance of global communication emerges under the condition that the value of *K* is equal to *N*-1. However, in real interactions of human beings, it is difficult to perform global communication. Thus, it is meaningful to analyze local communications of different degrees; in our model, the degree can be represented by *K*. In addition, the authors also defined a global communicator to research mixed communication and discovered that a few globally communicating individuals can steer outcomes toward their preferences, although there is a weakening effect from all other individuals that communicate locally with their neighbors [10]. These global communicators can be described as leaders in the group. We defined the communication ability of an individual by the fixed number of nearest neighbors from whom they can obtain information, which is determined by the value of *K* and is equal to the value of the in-degree. However, the ability to affect other individual is not limited, which is represented by the value of the out-degree. This also illustrates that the individuals in our model are not completely homogeneous. The results show that a leader emerges in the process of pursing the maximum benefits, and this implies the significant role of the leader, who is able to drive all other individuals to move toward a common goal. This is surely a kind of mechanism in the process of the emergence of collective behavior in a group.

In psychology, confirmation bias is the tendency of people to overestimate the probable accuracy of their opinions; that is, people assess the merits of their own opinions partially and are unwilling to approve of and accept the opposite opinions from other people [38]. Moreover, the reason that a large group would split up is that when individuals in the group are too far away from each other to communicate, they only considered those in their immediate surroundings [9]. Therefore, the results of our model are consistent with these phenomena, which are based on the rules of topological interactions among individuals (the rules of nearest neighbors). However, we consider another situation that occurs in real human society: an isolated person who far from all other people is more willing to move toward the crowd. Sociality makes people afraid of being isolated, and people hope to be respected and loved by other people [39]. This phenomenon cannot be explained by the rules of interactions among individuals based on metric distance. Because the perception radius of an individual is limited, the isolated individual will remain still as he cannot perceive other individuals or obtain information from them. The rules of topological interactions among individuals are more suitable for explaining this phenomenon. It is noted that the initial positions of individuals are set up characteristically; that is, there is an isolated individual in the initial state. The aim is to test whether the isolated individual will get together successfully with the other individuals in the process of the emergence of collective behavior in the group. The results confirm the effectiveness of this method. Moreover, to represent the psychological phenomenon that people are afraid of being isolated, we assume that the attraction in Eq (1) will attain the maximum value when the distance between two individuals exceeds a threshold value. This also enables the model to simulate a kind of psychological state in which people always consider the opinions of the majority to be reliable and correct. Therefore, people are likely to change their opinions or behavior in accordance with those of the majority. In [11], Moussaïd et al. identified two major attractors of opinion that can affect public opinion formation: the expert effect and the majority effect. The former is caused by a highly confident and prestigious individual in the group, and the latter is caused by a great number of laypeople sharing similar opinions. One of these two attractors will dominate over the other one. Under this mechanism, collective opinions will emerge eventually. This also reminds us of the herding effect in the stock market. The herding effect can also lead to the snowball effect, as a number of traders prefer to mimic the major investment strategy or past trend when they are unable to distinguish which information is important [40]. That is, the expert effect and the majority effect also widely exist in the stock market. Herding behavior in the stock market is also a collective behavior that is based on the interactions among traders. However, this kind of collective behavior is regarded as harmful to the normal operation of the stock market. Bubbles and crashes in the stock market are more likely to emerge as the herding effect increases. Similar characteristics (nonlinear, chaotic and spiral characteristics) were also found in a study of the herding effect in our previous work [40]. Therefore, our simulations could allow us to determine the underlying mechanisms in the interactions of traders in the stock market, such as the prerequisites for the emergence of the herding effect and the methods to prevent or inhibit the harmful effects that come from this kind of collective behavior in the stock market. From the above, we discover that the rules of interactions among individuals in our model are suitable for explaining the interactions among human beings.

In this paper, we constructed a topological communication model based on a self-organization process. According to the results of simulations, we proposed explanations for the various types of collective behavior and fission behavior in the interactions of human beings. We discovered that the threshold of the emergence of collective behavior is five to seven and the number of neighbors that people are always interacting with may be within [5, *N*/2], which are the range for the number of topology *K*. Then, we analyzed the network properties of the model and found that the in-degree of individuals is always equal to the value of the number of topology. In the stable state, there is a common rule that governs the distribution of the out-degrees of individuals. The out-degrees of individuals distribute around the value of the number of topology *K*, except that the out-degree of a single individual is approximately double the out-degrees of the other individuals. In addition, under different initial conditions, some features of different kinds of networks (e.g., the *k*-regularity graph and small-world network) emerge from the model. To sum up, the results presented in this paper should be viewed as a new attempt to explain the rules of interactions in human behavior. Although this method has been shown to work for simulated swarms and various outcomes have occurred, its accuracy and suitability in practical circumstances need to be proven in future work. It may be difficult to describe the real rules in detail because human systems are highly complex. Nonetheless, we can keep trying to use the rules of various objects from different areas to explain the rules of human beings, as described by the concept of synergetics in [34], which proposed that there must be a common rule in various kinds of systems because many similar phenomena emerge in different systems. In future work, we plan to find more connections between the model and the real rules in the interactions of human beings, and we will try to determine whether some regularities of the number of topology *K* exist in real interactions in human society. Thus, we are currently involved in searching for additional outcomes of this model, and we hope our work will contribute to the understanding of the real rules of interactions in human systems.
